# Fluorescent tagged episomals for stoichiometric induced pluripotent stem cell reprogramming

**DOI:** 10.1186/s13287-017-0581-7

**Published:** 2017-06-05

**Authors:** Christopher E. Schmitt, Blanca M. Morales, Ellen M. H. Schmitz, John S. Hawkins, Carlos O. Lizama, Joan P. Zape, Edward C. Hsiao, Ann C. Zovein

**Affiliations:** 1 Cardiovascular Research Institute, University of California San Francisco, San Francisco, CA 94158 USA; 2 Eli and Edythe Broad Center of Regeneration Medicine and Stem Cell Research, University of California San Francisco, San Francisco, CA USA; 3 Division of Endocrinology and Metabolism, Institute for Human Genetics, Department of Medicine, University of California San Francisco, San Francisco, CA 94143 USA; 4 Division of Neonatology, Department of Pediatrics, University of California San Francisco School of Medicine, San Francisco, CA 94143 USA

**Keywords:** Induced pluripotent stem cells, Reprogramming, Pluripotent stem cells, Episomals, Yamanaka factors, OKSM

## Abstract

**Background:**

Non-integrating episomal vectors have become an important tool for induced pluripotent stem cell reprogramming. The episomal vectors carrying the “Yamanaka reprogramming factors” (Oct4, Klf, Sox2, and L-Myc + Lin28) are critical tools for non-integrating reprogramming of cells to a pluripotent state. However, the reprogramming process remains highly stochastic, and is hampered by an inability to easily identify clones that carry the episomal vectors.

**Methods:**

We modified the original set of vectors to express spectrally separable fluorescent proteins to allow for enrichment of transfected cells. The vectors were then tested against the standard original vectors for reprogramming efficiency and for the ability to enrich for stoichiometric ratios of factors.

**Results:**

The reengineered vectors allow for cell sorting based on reprogramming factor expression. We show that these vectors can assist in tracking episomal expression in individual cells and can select the reprogramming factor dosage.

**Conclusions:**

Together, these modified vectors are a useful tool for understanding the reprogramming process and improving induced pluripotent stem cell isolation efficiency.

**Electronic supplementary material:**

The online version of this article (doi:10.1186/s13287-017-0581-7) contains supplementary material, which is available to authorized users.

## Background

One of the major challenges in the creation of induced pluripotent stem cells has been the low efficiency of the reprogramming process. Since the initial findings that somatic cells can become pluripotent after enforced expression of Oct4, Sox2, Klf, and c-Myc [1], modifiers such as Lin28 and Nanog [2], p53 knockdown [3], and the substitution of L-Myc for c-Myc [4, 5] have been used to improve reprogramming efficiency from the initial reports [6].

Direct control and verification of the plasmid dosage taken up by a cell may be important for different aspects of reprogramming. The ratio of reprogramming factors has been shown to be important in reprogramming processes [7–9]. It was recently shown that high Oct4/Klf4 and lower Sox2/c-Myc created iPS colonies more efficiently and of a higher quality [10]. The higher quality iPS colonies are considered more ES-like and have been shown to perform better in tetraploid complementation and chimerism assays. One attribute of an ES-like state is the retention of expression at the Dlk1–Dio3 locus [11]. This imprinted region is often fully silenced in iPS cell clones. Genes within this locus have been shown to be differentially expressed when comparing ES with iPS cell colonies [11, 12]. It is therefore desirable to directly recover iPS cell colonies that are ES-like in reprogramming efforts.

Although there are a large number of reprogramming systems available, the use of fluorescent reporters to mark individual reprogramming factors has not been applied widely. In this study, we tailored the Yamanaka episomal vectors (Oct3/4, Klf4, Sox2, L-Myc + Lin28. and p53 shRNA) [5] to also express separable fluorescent proteins. This strategy allows for direct assessment of plasmid dosage and for the sorting of successfully transfected cells for improved programming. In addition, it provides a tool to track episomal expression in real time through cell imaging of the surrogate fluorescent proteins.

## Methods

### Cloning of tagged episomal plasmids

Fluorescent protein expression cassettes were generated using PCR (NEB Phusion) and restriction enzyme-mediated cloning. From original plasmid vectors, the single *Cla*I site in the vector backbone was modified into a *Cla*I-*Fse*I-*Asi*SI-*Cla*I multiple cloning site. The Sox2 and Klf4 coding sequences from the original Sox2-2A-Klf4 sequence were separated by PCR (NEB Phusion), adding flanking *Eco*RI sites and a new stop codon for Sox2.

#### Modification of pCXLE-hOCT3/4-shp53-F (Addgene 27077) and pCXLE-hUL (Addgene 27080) vectors (L-myc/Lin28)

To generate a new multiple cloning site (MCS), oligonucleotides 5′-*agatcgcgatcgcagggccggccatcgatag*-3′ and 5′-*ctatcgatggccggccctgcgatcgcatcgatct*-3′ were annealed and then restriction digested with *Cla*I restriction enzyme (New England Biolabs). The *Cla*I-digested MCS insert was then purified by NaOAc precipitation. Plasmid vectors were digested with *Cla*I, treated with alkaline phosphatase (New England Biolabs), and gel-purified using the Qiagen Gel extraction kit. The MCS insert and plasmid backbone was then treated with T4 DNA ligase (New England Biolabs). The resulting ligation reactions were then transformed using Top10 competent cells (Life Technologies) and selected with ampicillin. The resulting colonies were mini-prepped (Qiagen) and screened for linearization by *Fse*I and *Asi*SI enzymes (New England Biolabs). The resulting intermediate plasmids were termed “Oct4/p53 + FA MCS” and “L-myc/Lin28 + FA MCS”.

#### Generation of pCXLE-hOCT3/4-shp53 + CMV:mCherry-2A-Puro (Addgene 27080) and pCXLE-hUL + CMV:mTAGBFP2 (Addgene 54572) vectors

The expression cassette of CMV:mCherry-2A-Puro was PCR amplified with addition of *Fse*I and *Asi*SI (New England Biolabs) restriction sites. The CMV:mCherry-2A-puro expression cassette was then directionally cloned into the Oct4/p53 + FA MCS vector. For the L-Myc-2A-Lin28 mTagBFP2 vector, *Fse*I and *Asi*SI sites were also used to directionally insert a CMV:mTAGBFP2-bGHpA expression cassette.

#### Generation of Sox2 + CMV:eGFP and Klf4 + CMV:E2Crimson (Addgenes 27078 and 38770) vectors

The pCXLE-hUL + CMV:mTAGBFP2 vector was digested with *Eco*RI to remove the Lin28-2A-L-Myc ORF and was replaced with either Sox2 or Klf4 PCR-generated ORFs. After amplification and sequencing of the vectors, they were then cut with *Xho*I and *Age*I (New England Biolabs) to remove the mTAGBFP2 ORF and replaced with either E2-Crimson or eGFP, generated by PCR with compatible ends.

### Transfection

The modified plasmids were purified using the Qiagen Endotoxin-free Maxi-prep kit. Transfection into human foreskin fibroblasts (UCSF cell culture facility, catalog number CCLZR211, log number MB3145, passages 9–20) was performed using the Neon Transfection system as described previously [13]. Lines carrying the transfected factors were designated with O (Oct3/4 + shp53), K (KLF4), S (Sox2), or M (L-Myc + Lin28) respectively, using uppercase or lowercase to designate high or low fluorescence. After electroporation, cells were plated onto Bovine bovine collagen I (Corning)-coated dishes in recovery medium (DMEM H21 with 10% FBS without antibiotics). Medium was changed the next day to DMEM with 10% FBS and 1× penicillin/streptomycin for continued culture into iPS cells. After 4 days, cells were gradually transitioned to mTesR iPS cell media as described previously [13].

### Cell culture

All iPS cell lines showed normal karyotyping or comparative genomic array analysis (Cell Line Genetics). iPS cells were maintained on feeder cells (SNLs) with hESC medium (Knockout DMEM, 20% Knockout Serum Replacement, 1× sodium pyruvate, 1× non-essential amino acids, 1× Glutamax, 0.5× penicillin/streptomycin solution, 0.1 mM 2-mercaptoethanol) supplemented with 10 ng/ml bFGF as described previously [13]. On passage 11 the cells started their transition to mTesr1 with increasing ratios of hESC medium:mTesr1: 1:3, then 1:1 on passage 12, 3:1 on passage 13, and finally feeder-free conditions on passage 14 on Matrigel-coated plates and mTesr1 media (StemCell Technologies, Vancouver, Canada). Every time the cells were split, the medium was supplemented with 10 μM Y-27632. Embryoid bodies for assessment of differentiation capacity were generated as described previously [13]. All iPS work was approved by the UCSF Human Gamete, Embryo, and Stem Cell Research Committee (GESCR) and the UCSF Committee for Human Research.

### Cell lines

Human foreskin fibroblasts were sourced from the UCSF cell culture facility (catalog number CCLZR211, log number MB3145, passages 9–20). The BJ2 wildtype iPS cell line, previously generated from BJ foreskin fibroblasts commercially available from ATCC (catalog number ATCC CRL-2522), were used as a control [13].

### Embryoid bodies

Embryoid bodies were generated as described previously [13]. The EBs were maintained until day 15 and harvested for RNA extraction in TriReagent (Sigma Aldrich).

### Teratoma formation

iPS cells were grown on Matrigel-coated plates with mTesr1 until reaching 90% confluency. The cells were injected into the testes of CB-17/SCID mice (Charles River) as described previously [13]. Tumors were harvested 8–12 weeks after the procedure. Three teratomas for OKSM and four teratomas for OKSM NE were analyzed.

### Flow cytometry

Human foreskin fibroblast lines transfected with the plasmids were sorted on a BD FACS Aria3 at the lowest flow rate of “1” with a 130-μM sort nozzle to maintain viability.

### OKSM sorting strategy

**Fig. 1:**
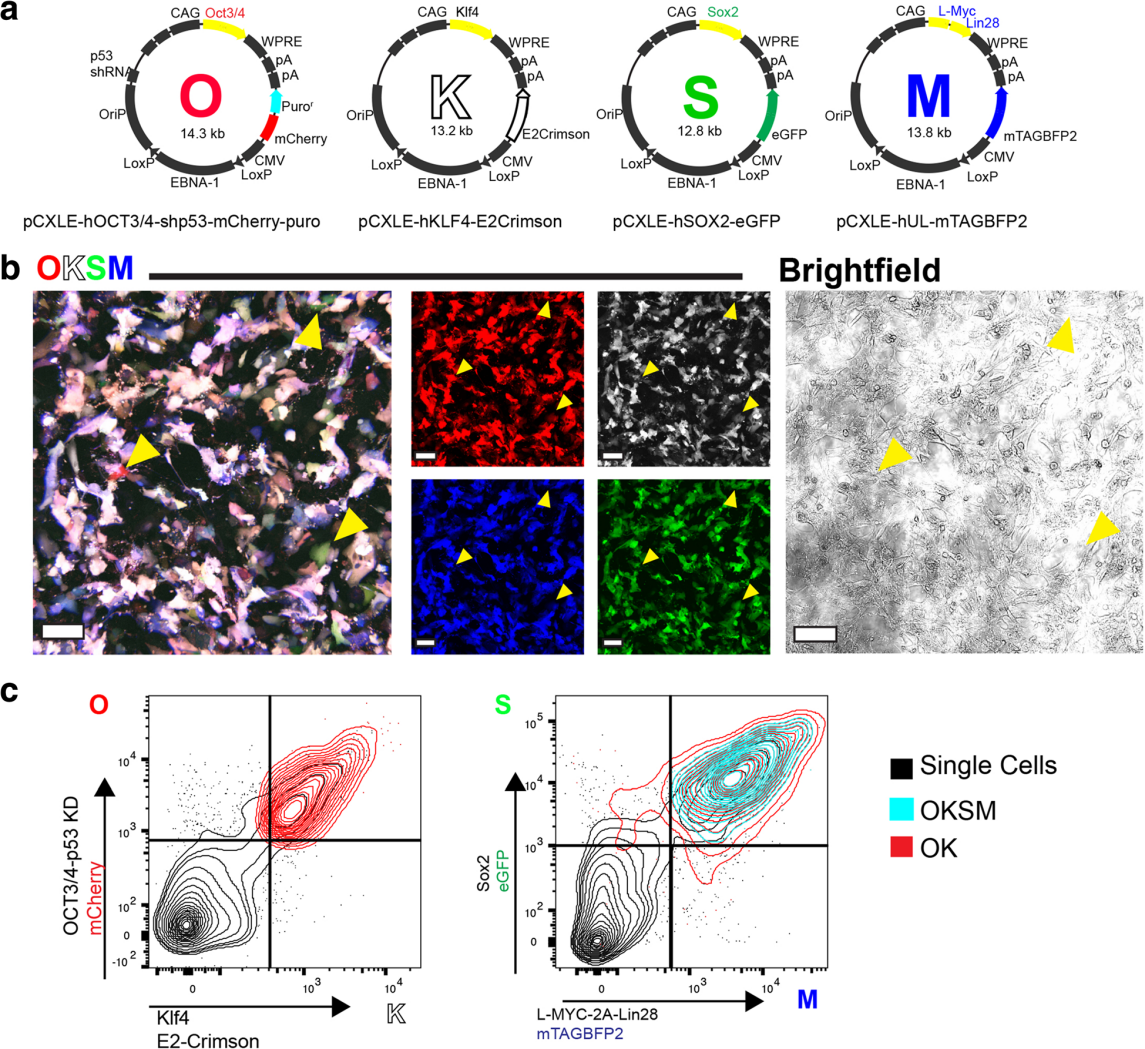
Fluorescent tagged episomal vectors allow for enrichment of OKSM-positive cells. **a** Schematic of engineered reprogramming episomal plasmids. Fluorescent protein expression cassettes were inserted downstream of the reprogramming factor expression cassettes, indicated by color. **b** Tiled laser-scanning confocal maximum projections capture the heterogeneity of episome-carrying cells. Confluent human foreskin fibroblasts at 2 days post transfection of plasmids are shown. *Arrowheads* indicate cells with discrepant levels of fluorescent proteins, significantly favoring expression of one episome over another. *Scale bars* = 150 μM. **c** FACS discrimination of the OKSM population. *Left*: whole population (*black*) gated for mCherry (Oct 3/4) (*O*) and E2-Crimson (Klf4) (*K*). The double-positive population (*OK*) is shown in Quadrant 2 (*red*). Right: whole population (*black*) and OK (*red*), subsequently gated for eGFP (Sox2) (*S*) and mTAGBFP2 (L-Myc-2A-Lin28) (*M*). *Cyan* contours designate the OKSM population (Color figure online). *OKSM* Oct3/4 + shp53, Klf, Sox2, and L-Myc + Lin28

The original sorting strategy was to select cells positive for all four episomals (OSKM) per traditional gating depicted in Fig. 1c.

In order to designate high (OKSM) vs low (oksm) fluorescent expression in our transfected populations (Figs. 3, 4), the median fluorescence value (MFV) of each fluorophore was calculated using BD FACSDiva software. The positive-gated population for each fluorescent protein was used to calculate the respective MFV. To then determine the high vs low positive populations, the coefficient of variation (CV%) of the positively gated population (automatically calculated by BD FACSDiva software) was calculated. The CV% is the ratio of the standard deviation to the mean, which measures population dispersion. High and low fluorescence designations were then determined by setting gates outside a perimeter of 1% CV from the MFV. The bottom-left corner of the high gate and the top-right corner of the low gate were placed at (+1,+1) and (–1,–1), respectively, from the MFV. In this instance, the MFV is the center origin (0,0) and the units of the *X* and *Y* axes are CV% (Fig. 4b).

### hiPSC sorting strategy

For sorting of mature, passage 9+ iPS cells, Tra-1-60 (Stemgent) antibody was used in conjunction with DAPI for live/dead discrimination (see Additional file 1: Figure S4 for gating example). A total of 1000–2000 cells were sorted per iPS cell clone.

### Assessment of reprogramming efficiency

**Fig. 2:**
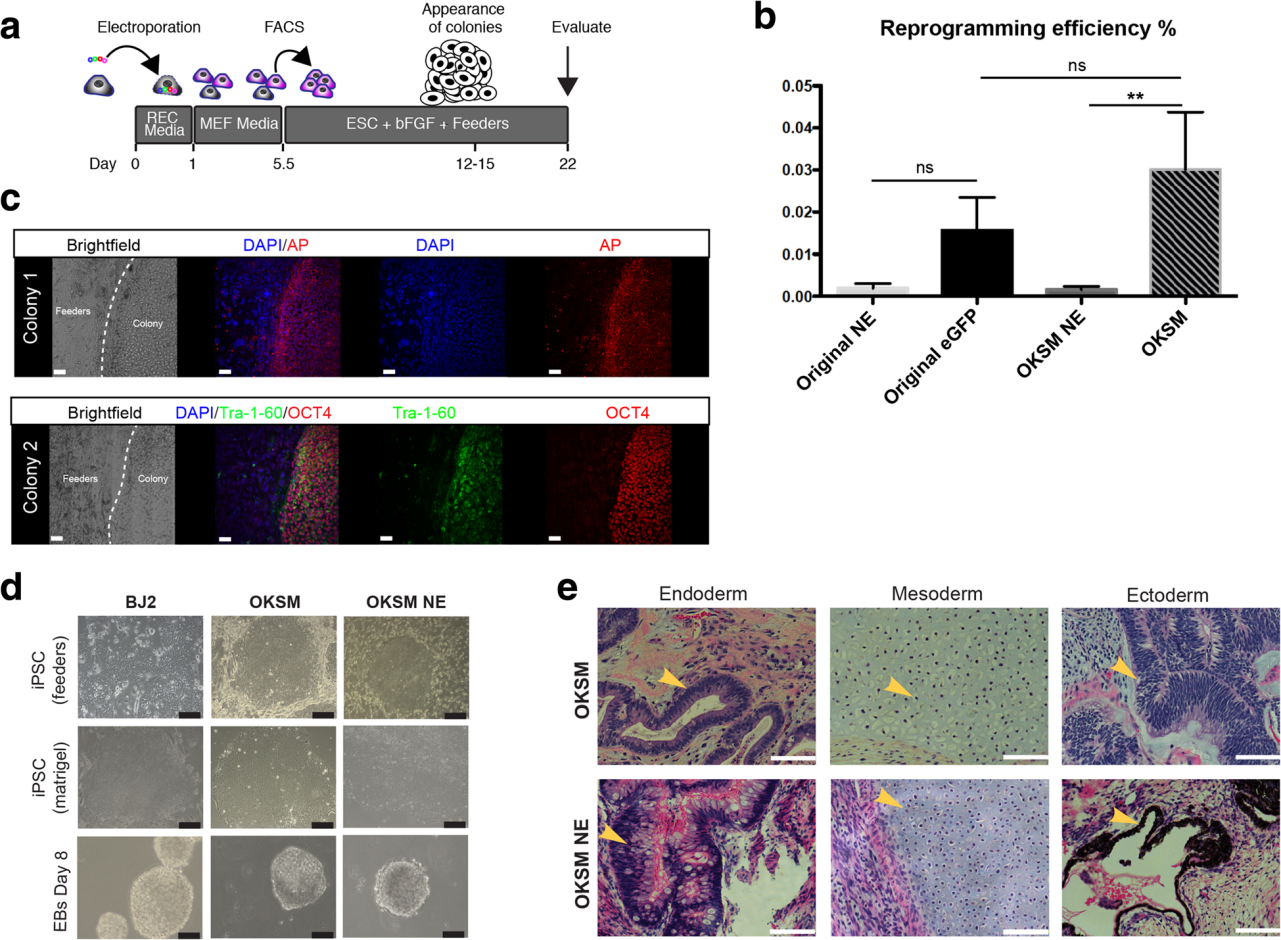
Enrichment of OKSM by FACS generates phenotypic iPS colonies. **a** Schematic for reprogramming with sorting enrichment, with the FACS enrichment occurring on day 5.5. **b** Reprogramming efficiency of sorted cells. *N* > 4 for each condition. *Original* unmodified Yamanaka episomal plasmids, *NE* non-enriched, *OKSM* cells reprogrammed using our tagged episomal vectors. *p* value determined by Mann–Whitney *t* test. Non-significant (*ns*) values left to right: *p* = 0.13, *p* = 0.37; **p = 0.002. **c** Immunohistochemistry for pluripotency markers on iPS cell colonies from quadruple-positive OKSM sorts showing positivity for markers: Oct3/4, alkaline phosphatase (*AP*), and Tra-1-60. Cells were stained at passage 8 (approximately day 60 post transfection). **d** Episomal-derived iPS cell embryoid body (*EB*) formation. iPS cells were transitioned from feeders to Matrigel and finally into EB differentiation media. *Top*: iPS cells on SNL feeder cells. *Middle*: iPS cells transitioned to feeder free conditions. *Bottom*: EBs at day 8 of differentiation. *Scale bar* = 200 μm. **e** Hematoxylin and eosin staining of teratoma sections showing representatives of the three germinal layers (*yellow arrows*). *Scale bar* = 200 μm (Color figure online). *iPSC* induced pluripotent stem cell, *OKSM* Oct3/4 + shp53, Klf, Sox2, and L-Myc + Lin28

Reprogramming efficiency was calculated as the number of iPS colonies at day 22 divided by the number of cells sorted/plated on day 5.5 (Fig. 2a), and expressed as a percentage.

### Gene expression analysis (qRT-PCR)

RNA was extracted using the Qiagen RNeasy Micro kit. cDNA was generated using the Superscript VILO Mastermix from Thermo Fischer. Primer sequences are presented in Additional file 1: Table S1. For copy number quantitation, a 10-fold dilution series of the appropriate plasmid was used to correlate copy number to cT and also used to calculate the efficiency of the primers [13]. Each reaction was run in triplicate on a Viia7 Real-Time PCR System (Life Technologies) and normalized to GAPDH as an endogenous control.

### Immunocytochemistry

Vector Red alkaline phosphatase staining kit (Vector Labs) or Live AP (Life Technologies) at 1:100 dilution; Stainalive Tra-1-60 antibody (09-0068; Stemgent) at 1:200; and mouse monoclonal Oct3/4 antibody (sc-5279; Santa Cruz) 1:100 dilution with Alexa Fluor® 594 Donkey anti-mouse secondary at 1:300 dilution were used. Antibody incubations were performed at 4 °C. Cells were counter-stained with DAPI in PBS and imaged in PBS.

### Statistical analysis

Reprogramming efficiencies were compared using a *p* value determined by the Mann–Whitney *t* test. qPCR of OKSM and fluorescent proteins was analyzed by both two-tailed *t* test and ANOVA (see Additional file 1: Table S2). Fluorescence intensity (MFI) was compared with mRNA copy number via liner regression (*R* ^2^). Relative expression of MEG3 was measured via Student’s *t* test. *p* ≤ 0.05 was considered statistically significant.

## Results

### Fluorescence-facilitated identification of transfected populations

To allow identification of each key Yamanaka reprogramming factor [5], we first separated the Oct4 (+ shp53), KLF4, Sox2, and L-Myc (+ Lin28) open reading frames and added a fluorescent protein expression cassette. Vectors are designated by letters: (O) Oct3/4, shp53 and mCherry, (K) Klf4 and E2Crimson, (S) Sox2 and eGFP, and (M) L-Myc, Lin28, and BFP (Fig. 1a). The fluorescent proteins allow identification of cells that take up all four episomals, as well as cells that contain one, two, or three episomals, as observed by confocal microscopy (Fig. 1b). The tagged episomal set also allowed for cell sorting and enrichment of cells successfully transfected with all four episomal plasmids (OKSM; Fig. 1c; flow cytometric controls depicted in Additional file 1: Figure S1). Expression of the fluorophores was also possible in live cell cultures (Additional file 1: Figure S1E). Flow cytometric percentages of OKSM populations were similar regardless of the order of fluorescent selection by FACS (Additional file 1: Figure S2A). Both microscopy and flow cytometric analyses revealed that a significant number of cells express fewer than four plasmids after transfection (Fig. 1c).

### Fibroblasts receiving tagged episomes allow for FACS enrichment and increased efficiency of reprogramming

We compared the reprogramming efficiency of fibroblasts transfected with either our four tagged episomals or with the original untagged Yamanaka episomal set [14] consisting of three episomal plasmids containing programming factors and one episomal with an eGFP tracer. Cells were sorted on day 5.5 for all four fluorescent tags, or for the eGFP tracer alone. The isolated cells were then evaluated for ability to generate iPS cell-like colonies per previous protocols [14] (Fig. 2a). Unsorted cells run through a sorter but not selected/enriched for fluorescence (not enriched (NE)) were used as a control.

Quantitation of alkaline phosphatase (AP)-positive colonies (Additional file 1: Figure S2B) showed that the overall reprogramming efficiency of the MB132 foreskin fibroblasts, without FACS enrichment, were equivalent between the original Yamanaka plasmids and our tagged episomal plasmids, but lower than reported previously for other cell types. The introduction of FACS enrichment increased the efficiency of alkaline phosphate-positive colonies (trend when using GFP to enrich the original plasmids, vs statistically significant improvement when enriching for tagged cells containing all four OKSM factors). The use of tagged episomals resulted in a reprogramming efficiency of approximately 0.03% (Fig. 2b, Additional file 1: Table S1).

A subset of colonies generated by fluorescent selection of OKSM was evaluated at passage 8 (approximately 60 days post transfection) for pluripotency markers. These cells expressed Tra-1-60, AP, and endogenous Oct3/4 (Fig. 2c). In addition, colonies generated from sorted OKSM formed all three germ layers in embryoid body formation (Fig. 2d) and in vivo teratoma assays (Fig. 2e). The generated iPS cells and EBs were evaluated via qPCR, which validated iPS pluripotent gene expression and germ layer gene expression after differentiation (Additional file 1: Figure S3).

### Fluorescent tagged episomals allow for sorting on factor dosage

**Fig. 3:**
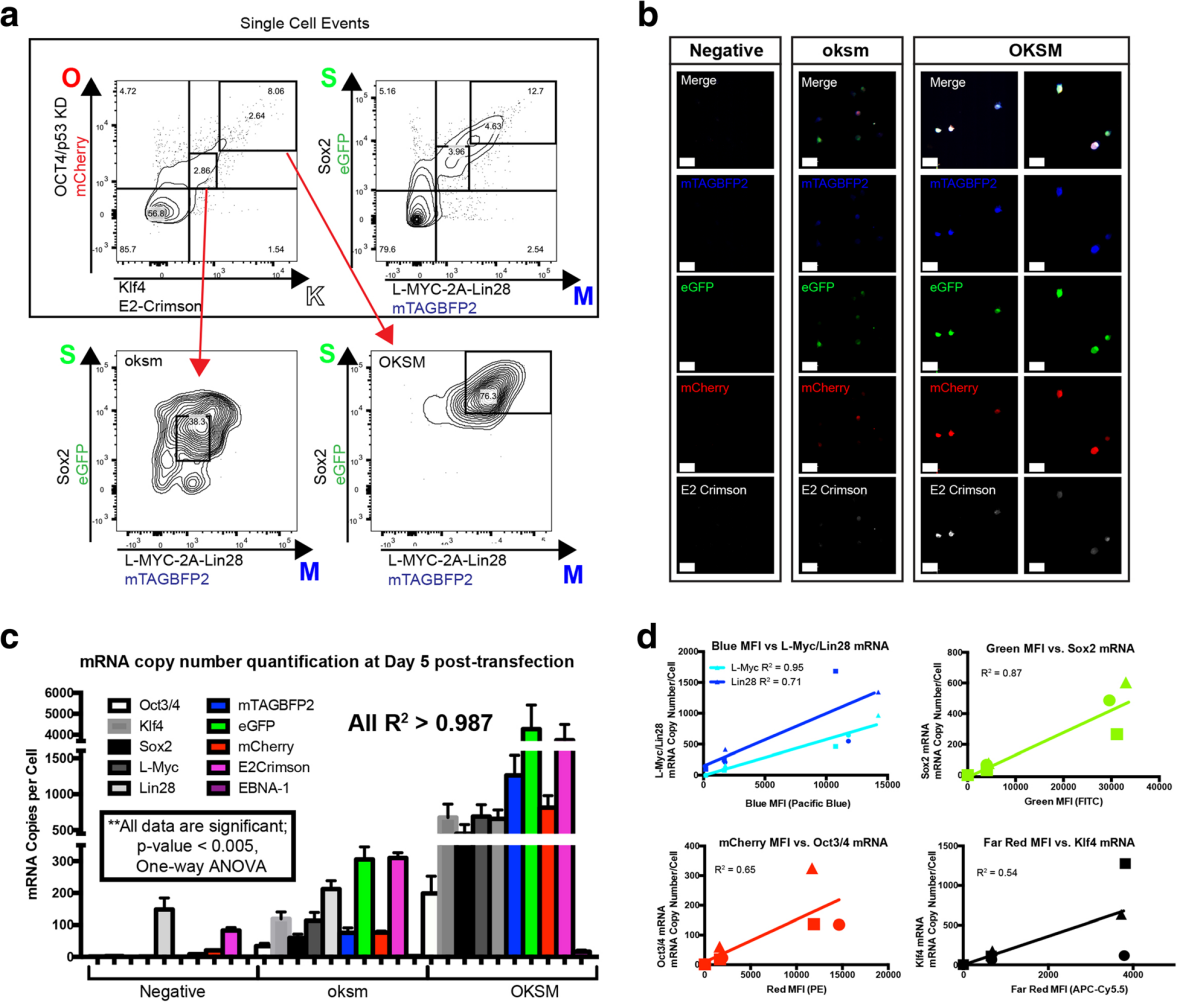
Fluorescence intensity correlates with reprogramming factor transcription levels, and allows for discrimination between high and low levels of O, K, S, and M. **a** Gating strategy for discriminating high vs low fluorescence. Single cells out of 10,000 events are shown. Gates are drawn according to the process described in Methods. The top two FACS plots show single events. The bottom two plots are the ok and OK populations (low vs high OK expression), respectively (*blue* vs *green* plots). Gates delineating the sm and SM populations are shown. Percentages shown are relative to the parental gates. **b** Laser-scanning confocal images of transfected HFFs after sorting for negative, low fluorescence (*oksm*), and high fluorescence (*OKSM*) on day 6 confirm the output of FACS. **c** Quantification of exogenous mRNA by qRT-PCR. HFFs sorted for negative, low, and high fluorescence were assayed by qPCR directly after sorting. Each target was significantly different between groups, *p* < 0.005*. Error bars* represent SEM. *R* ^2^ > 0.987 for all mRNA copy numbers vs mean fluorescence intensity values. **d** Mean fluorescence intensity (*MFI*) of individual sorts measured during flow vs mRNA copy number per cell measured by qRT-PR of corresponding Yamanaka/Fluorescent reporter sets. Each point represents one biological replicate (Color figure online). *OKSM* Oct3/4 + shp53, Klf, Sox2, and L-Myc + Lin28

We found a striking range of fluorescent intensities present in the positive population (OKSM), suggesting that individual cells take up a wide range of episome dosages. To understand the effect of dosage on reprogramming, we selected for cells exhibiting high or low levels of fluorescence and correlated the intensity of fluorescence to reprogramming factor expression. Populations were sorted into two subsets: one population with high levels of all four fluorescent proteins (designated OKSM); and one population with low but positive fluorescence as compared with controls (designated oksm; Fig. 3a).

Microscopy demonstrated that OKSM cells exhibited brighter fluorescence in all channels as compared with the dimmer oksm cells; unexpectedly, levels of each fluorescent protein varied per OKSM cell (Fig. 3b). Transcripts for each exogenous reprogramming factor were quantified in addition to fluorescent protein transcripts, and there was a significant difference in expression levels between OKSM, oksm, and negative populations (Fig. 3c). Together there was a high correlation of transcript expression to fluorescence. Gene expression was compared with the corresponding fluorescent protein mean fluorescence intensity (MFI; Fig. 3d), and showed less correlation among the three biological replicates per group. These populations showed a proportionate mRNA copy number per cell as estimated by qPCR (Fig. 3c, d).

### A high Oct3/4/Klf4 to Sox2/Myc ratio preserves expression of the Dlk1–Dio3 locus in human cells

**Fig. 4:**
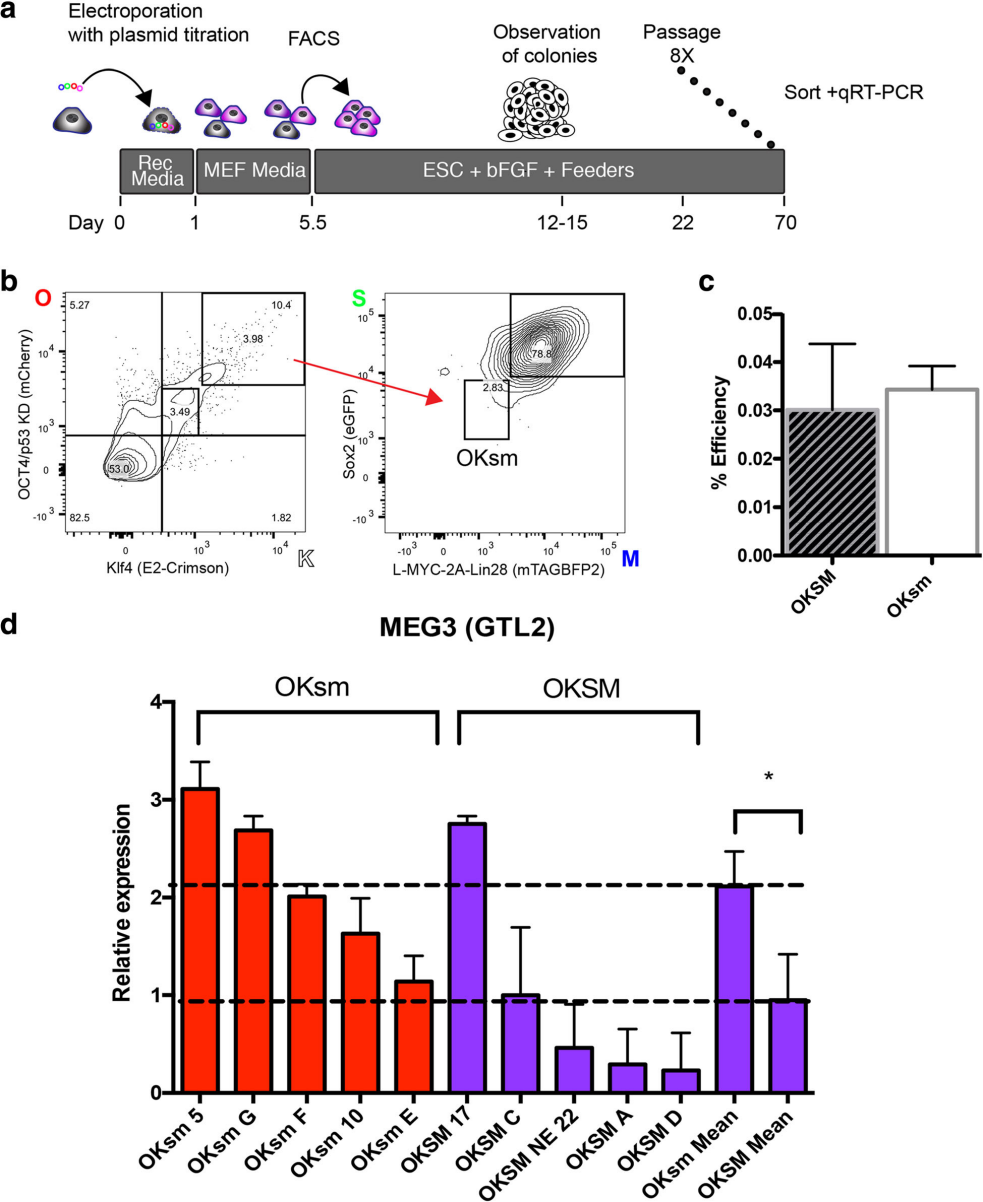
Reprogramming factor dosage selected via flow cytometry impacts iPS cell phenotype. **a** Schematic detailing procedure for isolating colonies reprogrammed with various plasmid dosages. As previously, cells are reprogrammed using our tagged episomal set. Plasmids were also titrated to favor the isolation of OKsm cells (see Methods). Prior to qRT-PCR, clones were passaged eight times to promote stable colonies. Sorting for the pluripotency marker Tra-1-60 and subsequent qRT-PCR for hMEG3 are subsequently performed. **b** Representative sort and gating strategy for isolating OKsm cells. Here, the ‘sm’ (*low blue* and *green*) population was 2.83% of the ‘OK’ (*high red* and *far red*) population or 0.11% of the total single events. **c** Reprogramming efficiency of OKSM vs OKsm sorted cells. *N* > 3, not significantly different *p* value. *Error bars* represent SEM. *p* = 0.15. **d**. Expression by qRT-PCR of the imprinted gene hMEG3 at passage 9 post reprogramming within the Tra-1-60+ population of cells (relative expression to *GAPDH*). Cells reprogrammed with OKsm (*red bars*) and OKSM (*purple*) are shown, each different clone designated by a letter or number. *Dashed lines* represent mean of each group. Clone names reflect the reprogramming method. *N* = 10 clones, *p* = 0.041*. Error bars* represent SEM (Color figure online). *OKSM* Oct3/4 + shp53, Klf, Sox2, and L-Myc + Lin28

The tagged episomal plasmids allow cells with negative, low, and high fluorescence to be purified. Because the fluorescence intensity correlates with reprogramming factor mRNA expression, we used our system to test whether cell populations with a high dosage of “OK” and low dosage of “SM” (designated OKsm; Fig. 4a, b) could still preserve expression of the Dlk1–Dio3 locus, because an ES-like state is correlated with Dlk1–Dio3 levels [11].

OKsm cells were less prevalent in the transfected population. We titrated the respective plasmids for collection of sufficient numbers of these cells. A hallmark of OKsm cells in the mouse is the retention of expression at the Dlk1–Dio3 locus [10] where the expression of gene *Gtl2* within the locus can act as a surrogate for locus imprinting [11, 12, 15]. We evaluated expression of the human homolog of murine *Gtl2*, MEG3. Sorted OKsm cells were able to reprogram at similar efficiency to OKSM (Fig. 4c). Colonies after multiple passages were sorted for Tra-1-60 (Additional file 1: Figure S4) and then evaluated for MEG3 (murine *Gtl2*) expression (Additional file 1: Tables S2–S4). OKsm colonies exhibited higher expression of MEG3 than OKSM colonies (Fig. 4d), indicating that the elevated OK to SM ratio has benefit for imprinting at the hallmark Dio3 locus in human cells.

## Discussion

Here we described a toolkit for the generation of integration-free human iPS cells. The original Yamanaka episomal factors [14] were revised to separate them, and fluorescent protein reporters were added. We found that these fluorescent proteins do not interfere with the reprogramming process, and allowed for real-time visualization of transfected cells, evaluation of transfection efficiency and heterogeneity, and the ability to enrich for populations with specific stoichiometry via cell sorting. Also, the fluorescent tagged episomals can allow for monitoring the status of all four episomes, and therefore present a viable method of screening out undesirable clones. While other groups have generated similar tools using lentiviral vectors [8, 16], our system provides traceable non-integrating episomal vectors. Although constitutive lentiviral vectors are silenced in hiPSC clones [8], they are known to leave a genomic “footprint” and demonstrate lower rates of aneuploidy as compared with episomals [17, 18]. Our study demonstrates an application of the individually marked episomal plasmids to select OKsm iPS colonies, and showed that these colonies maintained higher expression of MEG3, the human homolog of *Gtl2*, a gene within an imprinted locus that corresponds to ES cell-like iPS colonies with improved performance in pluripotency assays [10–12]. These results indicate that improved efficiency of iPS cell generation from human foreskin fibroblasts may be improved by a higher ratio of the OK to SM factors. In addition, the use of FACS purification may improve the yield of iPS cells by decreasing the number of cells that pick up one, two, or three of the plasmids, rather than all four.

## Conclusion

The fluorescent tagged episomal vectors allow for non-integrative iPS reprogramming while allowing for controlled Yamanaka factor stoichiometry. The system also gives the ability to monitor episomal expression in live cells by surrogate fluorescent detection. These new fluorescent-tagged episomal plasmids will useful to the stem cell community for improved selection and monitoring during iPS programming.

## Additional file

Additional file 1: presents supplementary figures and tables. (DOCX 3644 kb)

## Abbreviations

CV: Coefficient of variation
EB: Embryoid body
hESC: Human embryonic stem cell
hiPSC: Human induced pluripotent stem cell
iPS: Induced pluripotent stem
OKSM: Oct3/4 + shp53, Klf4, Sox2, and L-Myc + Lin28
