# Supplementary material for: Fluorescent tagged episomals for stoichiometric induced pluripotent stem cell reprogramming
Source: Stem Cell Res Ther. 2017 Jun 5;8:132. doi: 10.1186/s13287-017-0581-7 (PMC5460403; doi:10.1186/s13287-017-0581-7)
Supplement: Additional file 1: — presents supplementary figures and tables. (DOCX 3644 kb) [file 13287_2017_581_MOESM1_ESM.docx]

**
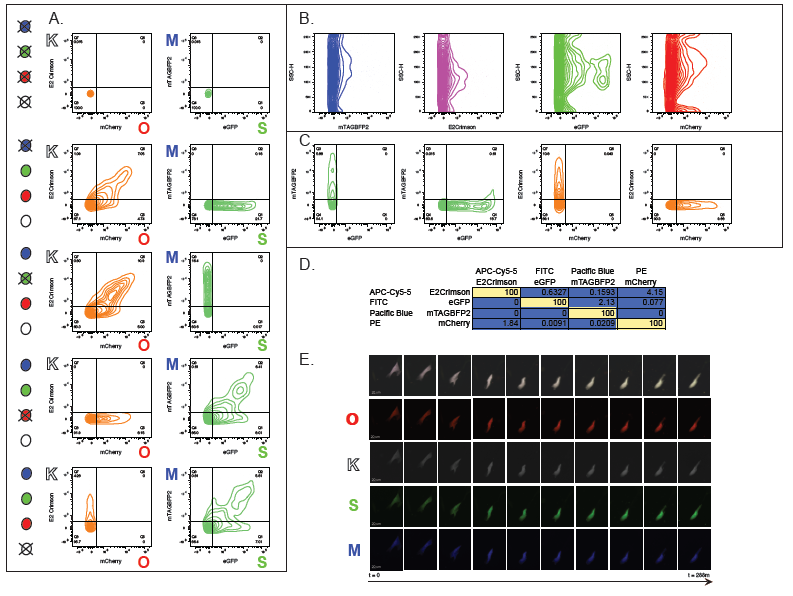
­­**

**Figure S1:** A. Flow cytometry controls of HFF cells transfected with either no plasmids (top) or a combination of 3 out of 4 plasmids with corresponding fluorescence (all minus one controls). B. Single color plasmid controls for each single fluorescent protein. C. Flow cytometry controls of HFF cells transfected with a single plasmid of each fluorescent color. D. Typical compensation matrix for sorting cells. E. Live imaging of a single cell transfected with all 4 plasmids exhibiting all four fluorescent proteins associated with O, K, S, M at 32 min intervals for a total of 288 mins, scale bar as shown. Top panel is merged, other panels are single color channels designated with corresponding plasmid (O,K,S,M).

**
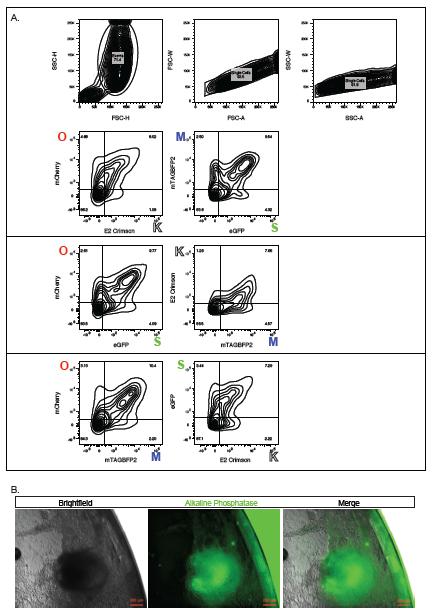
**

**Figure S2:** A. Flow cytometry gates for HFF cells transfected with all 4 plasmids gated on O, K, S, M, in various combinations. B. Live AP (Alkaline Phosphatase) staining of an iPS colony derived from transfected cells sorted for expression of all 4 plasmids, scale bar as shown.


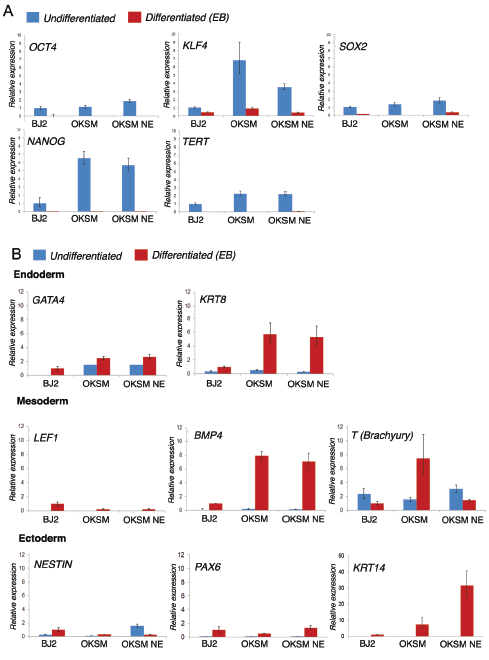


**Figure S3:** A. Quantitative PCR gene expression analysis showing higher expression of pluripotency genes in episomal iPS cells (blue) in comparison to EB differentiated iPS cells at day15 (red). B. Quantitative PCR gene expression analysis showing higher expression of the different germ layers (as labelled – endoderm, mesoderm, ectoderm) after 15 days of EB differentiation (red), compared with undifferentiated iPS cells (blue). Error bars are average expression +/− 1 SD of technical triplicates. Gene expression studies were repeated in triplicate. Gene expression normalized to *GAPDH* and relative to BJ2 iPS cells, OKSM and OSKM sorted but not enriched (NE).

A.A.


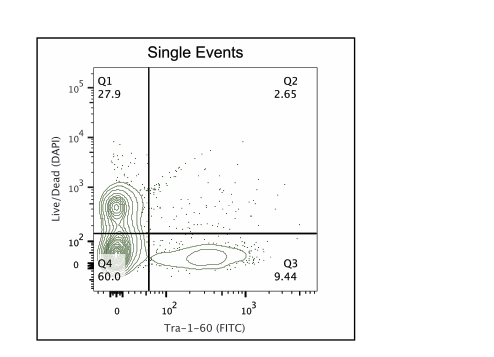


**Figure S4:** A. FACS plot of single events (singlet discrimination not shown). The x-axis is Stainalive-488 Tra-1-60 and the y-axis is Live/dead discrimination. Live/Tra-1-60+ (Q3) cells were sorted for the qRT-PCR in Figure 4D.

**Table S1. Reprogramming efficiency statistics in Figure 2B and 4C.**

| Reprogramming efficiencies | | |  |  |  |
| --- | --- | --- | --- | --- | --- |
|  | Original NE | Original eGFP | OKSM NE | OKSM | OKsm |
| N | 4 | 6 | 8 | 9 | 4 |
| Mean | 0.001875 | 0.01554 | 0.001542 | 0.03009 | 0.0343 |
| Std. Deviation | 0.002394 | 0.01955 | 0.002174 | 0.04108 | 0.009776 |
| Std. Error | 0.001197 | 0.007982 | 0.0007688 | 0.01369 | 0.004888 |
| Sum | 0.0075 | 0.09326 | 0.01233 | 0.2708 | 0.1372 |
|  |  |  |  |  |  |

**Table S2. Statistical analysis of qPCR data in Figure 3C.**

|  | Two-Tailed T-Test | | Two-Way ANOVA | |  |  |
| --- | --- | --- | --- | --- | --- | --- |
|  | Negative/Low | Low/ High | Row Factor | Column Factor | One-Way ANOVA | |
| "Oct4" | 0.00627007 | 0.004717943 | P = 0.3425 | P = 0.0007 | 0.0004 |  |
| Sox2 | 0.001099332 | 0.004865705 | P = 0.3994 | P = 0.0008 | 0.0004 |  |
| Klf4 | 0.000678955 | 0.005992106 | P = 0.3922 | P = 0.0008 | 0.0004 |  |
| L-Myc | 0.002473139 | 0.002191725 | P = 0.3482 | P = 0.0002 | < 0.0001 |  |
| Lin28 | 0.008411081 | 0.000785833 | P = 0.4535 | P < 0.0001 | < 0.0001 |  |
| mTAGBFP2 | 0.001408985 | 0.000817033 | P = 0.4118 | P < 0.0001 | < 0.0001 |  |
| eGFP | 6.96466E-05 | 0.002912915 | P = 0.4328 | P = 0.0004 | 0.0002 |  |
| mCherry | 7.37373E-06 | 0.000680222 | P = 0.4570 | P < 0.0001 | < 0.0001 |  |
| E2 Crimson | 1.15188E-05 | 0.000635882 | P = 0.4665 | P < 0.0001 | < 0.0001 |  |
| EBNA-1 | 0.043944664 | 0.005988137 | P = 0.3688 | P = 0.0017 | 0.0011 |  |

**Table S3. Taqman primer list used for quantitative PCR for iPS colonies and EBs**

| **GAPDH** | **Hs02758991_g1** | Glyceraldeyde-3-phosphate dehydrogenase | Control gene |
| --- | --- | --- | --- |
|  |  |  |  |
| **Pluripotency Markers** | |  |  |
| KLF4 | Hs00358836_m1 | Kruppel-Like Factor 4 | Transcriptional regulation of pluripotent stem cells |
| Nanog | Hs02387400_g1 | Homeobox Transcription Factor Nanog | Stem cell proliferation, renewal and pluripotency |
| OCT4 | Hs00999634_gH | POU Class 5 Homeobox 1 | Stem cell pluripotency |
| TERT | Hs00972656_m1 | Telomerase Reverse Transcriptase | Stem cell self-renewal and proliferation |
| Sox2 | Hs01053049_s1 | SRY-Box 2 | Embryonic development |
|  |  |  |  |
| **Differentiation Markers** | |  |  |
|  |  |  |  |
| **Endoderm** |  |  |  |
| Gata4 | Hs00171403_m1 | GATA binding protein 4 | Human endoderm marker |
| KRT8 | Hs02339473_g1 | Keratin 8 | Human endoderm marker |
| **Mesoderm** |  |  |  |
| BMP4 | Hs00370078_m1 | Bone morphogenic protein 4 | Human mesoderm marker |
| Lef 1 | Hs01547250_m1 | Lymphoid enhancer binding factor 1 | Human mesoderm marker |
| T | Hs00610080_m1 | Brachyury Transcription Factor | Human mesoderm marker |
| **Ectoderm** |  |  |  |
| Nestin | Hs00707120_s1 | Nestin | Human ectoderm (brain and eyes development) marker |
| KRT14 | Hs00265033_m1 | Keratin14 | Non-neuronal human ectoderm marker |
| Pax6 | Hs00240871_m1 | Paired box 6 | Human ectoderm marker |

**Table S4. Oligonucleotides used for Sybr-green qRT-PCR expression analysis experiments.**

| Target | Plasmid/  Target | Forward primer | Reverse primer |
| --- | --- | --- | --- |
| *hOct3/4* | OmCH | 5’- tttccccctgtctctgtcac | 5’- gactagtccccgaagcttga |
| *hSox2* | SG | 5’- gcaacgtgctggttattgtg | 5’- ttcagctccgtctccatcat |
| *hKlf4* | KCrim | 5’- cgccttacacatgaagagaca | 5’- aattttgtaatccagaggttga |
| *hL-Myc* | LLBFP | 5’- ggctgagaagaggatggctac | 5’- tttgtttgacaggagcgacaat |
| *hLin28* | LLBFP | 5’- agccatatggtagcctcatgtccgc | 5’- Tagcgtaaaaggagcaacatag |
| *mTAGBFP2* | LLBFP | 5’- ttcttagcgggtttcttgga | 5’- ctggaaggcagaaacgacat |
| *eGFP* | SG | 5’-agaacggcatcaaggtgaac | 5’-tgctcaggtagtggttgtcg |
| *mCherry* | OmCH | 5’-caagtagtcggggatgtcgg | 5’- aagctgaaggtgaccaaggg |
| *E2-Crimson* | KCrim | 5’-ccagttctacggctcca | 5’-ttgaggtagtcggggatgtc |
| *EBNA-1* | All | 5’-gaactgcccttgctattcca | 5’-gcatccttcaaaacctcagc |
| *GAPDH* | Human | 5’- ccactcctccacctttgac | 5’- accctgttgctgtagcca |
| *hMEG3* | Human | 5’- acggcggagagcagagag | 5’- tgatgtcatccctgaggagag |
| *hMEG8* | Human | 5’- attctttcttgcaccgatgg | 5’- cccagatccccttgaaaga |
